# Supplementary material for: Management of acute kidney disease as part of routine clinical care in low-resource settings: The International Society of Nephrology Kidney Care Network Project
Source: PLoS One. 2025 Apr 21;20(4):e0315802. doi: 10.1371/journal.pone.0315802 (PMC12011220; doi:10.1371/journal.pone.0315802)
Supplement: S1 Table — (DOCX) [file pone.0315802.s001.docx]

**Supplementary Table 1:** Study sites for KCN project implementation

|  | BOLIVIA | BRAZIL | NEPAL | SOUTH AFRICA |
| --- | --- | --- | --- | --- |
| Healthcare Centre (HCC) | CNS Comprehensive Family Medicine Center Quillacollo;  CNS Comprehensive Health Center Punata;  CNS Comprehensive Health Center Sacaba | Arapixuna HCC;  Tiningu HCC;  Alter do Chão HCC | Nil | Ezimpondweni clinic;  Hluhluwe clinic;  Mabibi clinic;  Manaba clinic;  Mbazwana clinic;  Mnqobokazi clinic;  Oqondweni clinic. All KwaZulu-Natal. |
| District Hospital | Hospital Central De Ivirgarzama;  Hospital Dr. Aurelio Melan Totora;  Hospital Municipal Autónomo de Colcapirhua | Hospital Municipal de Santarem | Nil | Mseleni Hospital, KwaZulu-Natal* |
| Tertiary Hospital | Hospital Obrero No 2, Cochabamba | Nil | B,P.Koirala Institute of Health Sciences, Dharan | Nil |

*not used as recruitment site, but follow-up undertaken here
