# Supplementary material for: Management of acute kidney disease as part of routine clinical care in low-resource settings: The International Society of Nephrology Kidney Care Network Project
Source: PLoS One. 2025 Apr 21;20(4):e0315802. doi: 10.1371/journal.pone.0315802 (PMC12011220; doi:10.1371/journal.pone.0315802)
Supplement: S2 Table — (DOCX) [file pone.0315802.s002.docx]

**Supplementary Table 2**: Definitions of kidney disease

| **KIDNEY DISEASE** | | |
| --- | --- | --- |
| **Kidney Disease** | **Definition** | **Comments** |
| ***AKD with AKI*** | Increase in SCr by 50% within 7 days  OR  Increase in SCr by 0.3mg/dl within 2 days from baseline | Stage 1: SCr increase by 1.5-1.9 times baseline;  Stage 2: SCr increase by 2.0-2.9 times baseline;  Stage 3: SCr increase by ≥3 times baseline or increase in SCr to ≥4mg/dl or initiated on KRT  Urine output criteria not used as data not captured |
| ***AKD without AKI*** | eGFR <60ml/min/1.73m^2^  OR  Decrease in eGFR by ≥35%  OR  Increase in SCr by 50% occurring over ≤3 months (but not within 7 days) | GFR estimated by CKD-EPI equation (2021)  Structural criteria (urinalysis) not used as data not captured as part of routine clinical practice |
| ***CKD*** | eGFR <60ml/min/1.73m^2^ for >3 months |  |
| ***NKD*** | Not fulfilling criteria for AKD or CKD |  |
| ***Baseline SCr*** | Latest creatinine documented prior to HCC/hospital admission  OR  Lowest creatinine during HCC/hospital admission | Lowest value of two criteria used  Imputed baseline creatinine based on an assumed eGFR not used |

AKD – Acute Kidney Disease; AKI – Acute Kidney Injury; CKD – Chronic Kidney Disease; NKD – No Kidney Disease; SCr – Serum creatinine; eGFR – estimated glomerular filtration rate; KRT – kidney replacement therapy.
