# Supplementary material for: Management of acute kidney disease as part of routine clinical care in low-resource settings: The International Society of Nephrology Kidney Care Network Project
Source: PLoS One. 2025 Apr 21;20(4):e0315802. doi: 10.1371/journal.pone.0315802 (PMC12011220; doi:10.1371/journal.pone.0315802)
Supplement: Attachment — Submitted filename: Response to reviews 7.11.24.docx [file pone.0315802.s005.docx]

We thank the reviewers, and the editorial team, for their feedback and helpful suggestions. Responses to each point are outlined below.

Journal Requirements:

When submitting your revision, we need you to address these additional requirements.

1. Please ensure that your manuscript meets PLOS ONE's style requirements, including those for file naming. The PLOS ONE style templates can be found at

https://journals.plos.org/plosone/s/file?id=wjVg/PLOSOne_formatting_sample_main_body.pdf and

https://journals.plos.org/plosone/s/file?id=ba62/PLOSOne_formatting_sample_title_authors_affiliations.pdf

The manuscript is formatted according to the above, to the best of our knowledge. Please reach out if further formatting changes are required.

2. Please include a complete copy of PLOS’ questionnaire on inclusivity in global research in your revised manuscript. Our policy for research in this area aims to improve transparency in the reporting of research performed outside of researchers’ own country or community. The policy applies to researchers who have travelled to a different country to conduct research, research with Indigenous populations or their lands, and research on cultural artefacts. The questionnaire can also be requested at the journal’s discretion for any other submissions, even if these conditions are not met.  Please find more information on the policy and a link to download a blank copy of the questionnaire here: https://journals.plos.org/plosone/s/best-practices-in-research-reporting. Please upload a completed version of your questionnaire as Supporting Information when you resubmit your manuscript.

This has been uploaded as Supporting Information, as requested. A section on ‘Inclusivity in global health research’ has been added also.

3. Thank you for stating the following in the Competing Interests section:

"I have read the journal's policy and the authors of this manuscript have the following competing interests: RE has received honoraria from Therakos. EB has received honoraria from Baxter and AstraZeneca."

This is stated.

Please confirm that this does not alter your adherence to all PLOS ONE policies on sharing data and materials, by including the following statement: ""This does not alter our adherence to  PLOS ONE policies on sharing data and materials.” (as detailed online in our guide for authors http://journals.plos.org/plosone/s/competing-interests).  If there are restrictions on sharing of data and/or materials, please state these. Please note that we cannot proceed with consideration of your article until this information has been declared.

This has been added.

Please include your updated Competing Interests statement in your cover letter; we will change the online submission form on your behalf.

This is included in the updated cover letter.

4. Your ethics statement should only appear in the Methods section of your manuscript. If your ethics statement is written in any section besides the Methods, please move it to the Methods section and delete it from any other section. Please ensure that your ethics statement is included in your manuscript, as the ethics statement entered into the online submission form will not be published alongside your manuscript.

The ethics statement has been moved to the main body of the manuscript.

5. Please include your tables as part of your main manuscript and remove the individual files. Please note that supplementary tables (should remain/ be uploaded) as separate ""supporting information"" files

Tables have been added to the main manuscript and separate supporting tables uploaded.

Reviewer #1: I thoroughly enjoyed reading this important study

Thank you for your support. We hope / anticipate the paper will be enjoyable to large parts of the PLOS ONE readership.

Introduction is clear. Aim is clearly defined
The KCN approach is nicely summarized.
How did they determine difference between AKI and AKD is recommended to be verbalized in the text.

Thank you for highlighting the need for clarity here. The distinction has been clarified from the outset (addition to the start of the introduction) and more details have also been provided in the ‘overview of the kidney care network approach section’.

Methods - Some clear description of article type is recommended. Is this a narrative summary?

This is a narrative summary – this is highlighted now at the end of the introduction.

Some clarity on difference between HCCs, district hospitals, and tertiary hospital is needed. I see it mentioned in the text eventually. But highlighting how this approach can be used in HCC would be strength

Thank you for this suggestion. The difference between facility types is highlighted in the first section of the project description. We agree that its potential to transform kidney care may be greatest in patients presenting to HCCs, and this has been specifically highlighted now in the first paragraph of the discussion.

What was the implementations strategy used? Perhaps a framework?

Thank you for this query. In each country, implementation of the approach was managed by project leads (SS, BC, EB, RDG), who were supported by the KCN leadership team (RE, KH, DH, MR) within the ISN. This detail has been added to the ‘overview of the KCN approach’ section, where further details of the strategy are outlined. Further details from each site are outlined in the country specific case studies.

Results and discussion
The team states “successful implementation of the KCN approach” – What were the parameters used to describe successful implementation?
The way I read the paper, it is a very descriptive summary evaluating feasibility of this approach. I would downplay the implementation aspect as implementation science approaches necessitates a more systematic evaluation of “success” parameters, fidelity etc.

Thank you for this excellent suggestion. We have rephrased our intentions and findings to focus more on feasibility, as suggested. This has been done in the first part of the discussion, as highlighted, but also in several other sections of the manuscript.

The summary from each country could benefit from a uniform and structured organization. scope, patients, etc.

A structured summary of the country level data is outlined in table 3.

I would highlight that this approach led to more female patients being screened.

Thank you for this important observation. The potential for the approach to promote more equitable care has been added to the first section of the discussion.

Some formatting comments
Would minimize use of abbreviations. There are many that are being used and the word is being spelt out again. For example, Page 7 line 215 KRT is spelt out again

This has been corrected and abbreviations are spelt out only once, at the time of first use.

Reviewer #2: This is a report on the findings following the implementation of a programme i.e. the Kidney Care Network project under the International Society of Nephrology's 0by25 initiative on reducing to zero number of the deaths that occur globally in people who would have developed acute kidney injury(AKI) which is supposed to be reversible in most instances. Most of these deaths have been occurring in resource limited settings. The cause of the AKI is usually secondary to conditions that are easily amenable to treatment with e,g just rehydration and treatment of sepsis. The authors claim to have implemented the project successfully but there are no data, even historical data, that the authors have shared in this article, for them to compare their current findings to. Thus there are no The manuscript is just a description/narrative of the findings in "4 healthcare centres across 3 continents".

Thank you for this summary. As highlighted in response to reviewer 1, we have clarified this paper to be a narrative summary. However, we feel this remains an important and unique addition to the literature. This is the first description of a protocol to manage AKD in LLMICs that has been embedded in routine clinical care – hence novelty in this regard. Moreover, we anticipate that the descriptive nature of the paper allows other centres to better appreciate the practicalities of implementation permitting the approach to be employed in diverse settings worldwide. The limitation of this being a purely descriptive paper has been added to the discussion (penultimate paragraph), and this limitation section also includes reference to the points raised below.

There is no definition of Acute Kidney Disease in this paper. The sampling strategy is not clear. Patients were screened for this renal dysfunction using a risk scoring system but we are also told that some were identified using a clinical assessment. Did the researchers include (screen) all patients presenting at these facilities where they were conducting this project? If not who was screened? Were those screened already "screened" clinical by the healthcare workers on the ground? Or were all consecutively seen patients screened? This explicit definition of this target population would allow the reader to be able to assess how these results can be "referred" back to the rest of the population.

Thank you for these comments. A definition of AKD is outlined in detail in S2 table. Moreover, we have clarified the distinction between AKD and AKI both at the start of the introduction (see response to reviewer 1), and also in the ‘overview of the kidney care network approach section’.

Given the large number of patients that present to the facilities included in the project, not all patients could be screened for practical reasons. Most patients were screened with the risk score, but inclusion for kidney disease testing was also permitted on clinical judgement alone, as the reviewer highlights. There may have been selection bias in terms of who was screened; moreover, we accept there was likely clinical heterogeneity in the clinical approach between sites, and this has been added to the discussion (penultimate paragraph / limitations).

The project was conducted in these 4 healthcare centres (HCC) in Bolivia, Brazil, Nepal and South Africa that were not similar. For instance in South Africa, the HCC was basically a primary HCC which one would expect not to to be able to have easy access to renal function tests and hence would have benefited from the POC testing for serum creatinine/educational activities being offered unlike the Nepal sites which was a higher level of care setting. We have no previous data to allow to judge if the findings of this project as presented were an improvement on the service delivery outcomes before this project came on board.

Thank you for highlighting the projects potential, specifically in lower levels of healthcare systems (ie. HCCs). This point was also highlighted by reviewer 1, and this has been emphasised in the first section of the discussion. In these settings, kidney testing was not previously available and as the reviewer suggests, the approach provides an obvious benefit at these locations.

The reviewer makes a good point in that the benefit may be less in higher levels of health systems. However, it is our experience that even in tertiary centres in LLMICs, kidney testing is often unavailable, and when it is, may come with significant delay. We are not able to provide specific data at the sites included in this project, beyond our anecdotal experiences working in these locations. However, published data from other low resourced sites highlights only the minority of patients have kidney functions tests taken during routine clinical practice^1^. Further anecdotal support of this comes from a previous audit of kidney disease management we previously undertook in unselected general medical admissions at a central hospital in Malawi; a decision to measure serum creatinine was made in 67.3% of patients, but this sample was only taken and processed in 53.3% and 21.3% of patients respectively (unpublished data). As such, we feel the strategy adds value even in the hospital setting in LLMICs, where timely assessment of kidney status is certainly not routine. We have added the above points to the discussion section (penultimate paragraph)

The authors refer to patients having been discharged but we are not told for instance for South Africa what this discharge meant. Were patients admitted and kept in the HCC for days as is likely to have been the case in perhaps Nepal a tertiary centre? These details need to be clarified.

Thank you for asking for clarification on this. The project included both patients who attended a healthcare facility, underwent serum creatinine testing (+- the relevant management) and were discharged on the same day (more likely in a HCC), in addition to those patients that were admitted and then discharged after an inpatient stay. ‘Discharged’ refers to both of these groups. This has been clarified in the project description section. Facility length of stay data were not collected and this has been added to the new section of the discussion that includes limitations.

The follow up of participants/ patients after 90 days does explain why the authors were using the term AKD as opposed to AKI.

The distinction and definitions of AKD and AKI have been clarified above, and within the manuscript itself.

This paper ideally should be reported using the following format: 1) Introduction 2) Project description 3) Project findings as opposed to using Discussion.

The paper has been reformatted as suggested. Thank you for this suggestion.

As currently presented, the manuscript is missing lots of detail in the project description which the author says is for another paper and not for this paper. The Discussion is just a narrative, The author refers to successful implementation of this project without summarizing the exact findings/results to support this assertion. What is the evidence for this statement? The educational activity was reported as having been conducted "regularly". What does this mean. There was no comparing or contrasting on this project findings to other findings in the wider literature.

The discussion has been majorly revised in response to the comments from both reviewers, as above. We have reframed the project to focus on feasibility of the implementation, as suggested by reviewer 1. The details of the educational activities are summarised in table 3.
